# Neuroprotective Effects of Serpina3k in Traumatic Brain Injury

**DOI:** 10.3389/fneur.2019.01215

**Published:** 2019-11-15

**Authors:** Yao Jing, Dianxu Yang, Yimu Fu, Wei Wang, Guoyuan Yang, Fang Yuan, Hao Chen, Jun Ding, Shiwen Chen, Hengli Tian

**Affiliations:** ^1^Department of Neurosurgery, Shanghai Jiao Tong University Affiliated Sixth People's Hospital, Shanghai, China; ^2^Department of Emergency, Shanghai Jiao Tong University Affiliated Sixth People's Hospital, Shanghai, China; ^3^School of Biomedical Engineering and Med-X Research Institute, Shanghai Jiao Tong University, Shanghai, China

**Keywords:** serpina3k, traumatic brain injury, mouse model, SH-SY5Y cells, apoptosis, oxidative stress, neuroprotection

## Abstract

Traumatic brain injury (TBI) is a major cause of disability and mortality worldwide, in part resulting from secondary apoptosis of neurons in peri-contusion areas. Serpina3k, a serine protease inhibitor, has been shown to inhibit apoptosis in injury models. In this study, we investigated the anti-apoptotic function of serpina3k *in vivo* using a mouse model of TBI, as well as the underlying neuroprotective mechanism *in vitro* using the SH-SY5Y human neuroblastoma cell line. TBI was induced in adult male C57BL/6 mice using controlled cortical impact. Serpina3k protein was intravenously administered at a concentration of 0.5 mg/kg twice daily for up to 14 days. SH-SY5Y cells were subjected to biaxial stretch injury and then treated with different concentrations of serpina3k. We found that endogenous serpina3k protein levels were elevated in peri-contusion areas of the mouse brain following TBI. Serpina3k-treated mice had fewer apoptotic neurons, lower levels of oxidative stress, and showed greater recovery of neurological deficits relative to vehicle-treated mice. Meanwhile, in the SH-SY5Y cell injury model, serpina3k at an optimal concentration (150 nM) inhibited the generation of intracellular reactive oxygen species, abrogated changes of the mitochondrial membrane potential, and reduced the phospho-extracellular regulated protein kinases (p-ERK)/ERK, phospho-P38 (p-P38)/P38, B cell lymphoma (Bcl)-2-associated X protein/Bcl-2, and cleaved caspase-3/caspase-3 ratios, thereby reducing the apoptosis rate. These results demonstrate that serpina3k exerts a neuroprotective function following TBI and thus has therapeutic potential.

## Introduction

Traumatic brain injury (TBI) is a major cause of disability and mortality worldwide (1, 2). In the United States, ~5.3 million people are living with TBI-induced disabilities (3). In China, the population-based mortality of TBI is estimated to be ~13 cases per 100,000 people (4). Irrespective of the outcome of these cases, the economic consequences of TBI are enormous (5, 6).

Primary injury following TBI leads to the death of numerous nerve cells in the damaged core regions. This is accompanied by apoptosis, programmed cell death, caused by secondary damage such as oxidative stress and inflammation around the core areas (7). Apoptosis occurs mainly in neurons, but also in astrocytes, oligodendrocytes, and endothelial cells from 6 h to 2 months post-injury, with the highest rates observed within 2 weeks (8). Therapeutic strategies targeting this reversible process of apoptosis can potentially improve TBI outcomes.

Serpina3k, also known as kallistatin, is a serine protease inhibitor with dual functions in apoptosis (9, 10): it has been shown to promote apoptosis in breast cancer cells (11) and colorectal cancer cells (12), while inhibiting apoptosis induced by oxidative injury in corneal epithelial cells (13) and retinal neurons (14). However, the function of serpina3k in the brain following TBI is unclear.

To address this point, the present study investigated whether serpina3k had neuroprotective effects following TBI using a mouse model, as well as the possible underlying mechanisms using a cellular model of experimental stretch injury (SI).

## Materials and Methods

### Animals

Adult male C57BL/6 mice (8–10 weeks old) weighing 20–25 g (Shanghai SLAC Laboratory Animal Corp., Shanghai, China) were used for experiments. Procedures involving mice were approved by the Institutional Animal Care and Use Committee of Shanghai Jiao Tong University.

### TBI Model

Mice were anesthetized using ketamine (75 mg/kg) and xylazine (10 mg/kg) and immobilized by placing the head in a stereotactic frame. A heating pad was placed under the mouse to maintain body temperature at 37°C. An ~10-mm long midline incision was made on the scalp under aseptic conditions; the skin and fascia were pushed aside and a 4-mm diameter bone window was drilled in the central aspect of the right parietal bone 1 mm lateral to the sagittal suture, with care taken to maintain the integrity of the dura; if this was damaged, the mouse was not used for experiments. All mice (including those in the sham-operated and TBI groups) underwent the surgical procedure. The TBI model was established using a controlled cortical impact (CCI) device (PinPoint Precision Cortical Impactor PCI3000; Hatteras Instruments, Cary, NC, USA). A 3-mm diameter rounded steel impactor tip was placed on the exposed intact dura and the cortical surface was hit vertically at an impact velocity of 1.5 m/s, deformation depth of 1.5 mm, and dwell time of 100 ms (15, 16). Bleeding of the injured cortical surface was controlled by applying a sterile cotton gauze and pressure. The cranial defect was sealed with sterile bone wax, and the incision was closed with interrupted 6-0 silk sutures. The animals were placed in heated cages until they regained full consciousness and then moved to their home cages.

### Western Blot Analysis of Brain Tissue

**Figure 1:**
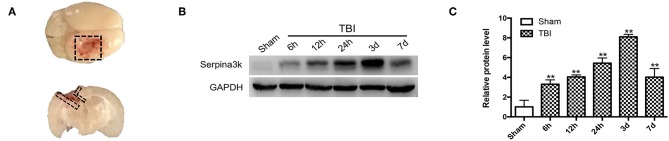
Serpina3k expressions before and after TBI in mice. **(A)** Black dotted areas showing the peri-contusion tissue region that was sampled for western blot and immunostaining. **(B)** Serpina3k protein expression before and at different time points after TBI, as determined using western blot. **(C)** Quantification of serpina3k levels from the immunoblotting experiment shown in panel B. GAPDH was used as a loading control. Data represent the mean ± SD (*n* = 5 per group). **P <0.01 vs. the sham group.

Brain tissue (Figure 1A) was collected from sham-operated and TBI mice at 6, 12, 24 h, 3, and 7 days post-surgery. The samples were lysed in radioimmunoprecipitation assay buffer (Millipore, Bedford, MA, USA) supplemented with a protease and phosphatase inhibitor cocktail (1:100; Cell Signaling Technology, Beverly, MA, USA) with 1 M phenylmethanesulfonylfluoride (Thermo Fisher Scientific, Waltham, MA, USA). The proteins were denatured and separated using sodium dodecyl sulfate–polyacrylamide gel electrophoresis, with equal amounts of protein loaded in each well. The proteins were transferred to a polyvinylidene difluoride membrane that was blocked with 5% skimmed milk powder and then incubated overnight at 4°C with primary antibodies against serpina3k (1:1000; Proteintech, Rosemont, IL, USA) and glyceraldehyde 3-phosphate dehydrogenase (GAPDH) (1:1000; Abcam, Cambridge, UK). After washing, the membrane was incubated for 1 h at room temperature with appropriate horseradish peroxidase-conjugated secondary antibodies (1:5000). Protein signals were detected using a gel imaging system (Millipore, Billerica, MA, USA) with enhanced chemiluminescence reagent (Pierce, Rockford, IL, USA), and signal intensity was analyzed using Quantity One software (BioRad, Hercules, CA, USA).

### Experimental Animal Design

Mice were randomly divided into three groups: sham-operated, and TBI with vehicle, or TBI with serpina3k treatment (TBI+vehicle and TBI+A3K, respectively). Recombinant serpina3k protein was obtained from Sino Biological (Wayne, PA, USA) and diluted with phosphate-buffered saline (PBS). Vehicle and serpina3k (0.5 mg/kg) solutions were intravenously administered straight after TBI and then twice daily until the end of the experiments. The dose of serpina3k was determined based on data from pilot experiments performed in our laboratory. Five animals per group were used for immunostaining detection and 12 for behavioral testing.

### Immunostaining

At 7 days post-surgery, brain tissue sections (Figure 1A) were collected from mice in the sham, TBI+vehicle, and TBI+A3K groups. Briefly, after fixing with 4% paraformaldehyde and 0.3% TritonX-100 and then blocking with 10% bovine serum albumin, the sections were incubated overnight at 4°C with the following primary antibodies: rabbit anti-NeuN (1:200; Millipore, Billerica, MA, USA), and mouse anti-3-nitrotyrosine (NT) (1:200; Abcam, Cambridge, UK)/rabbit anti-NeuN double-staining. Subsequently, they were incubated with TUNEL buffer (*in situ* Cell Death Detection Kit, TMR Red; Roche, Diagnostics, Basel, Switzerland) and corresponding secondary antibodies (1:500) for 1 h, then stained with 4′,6′-diamidino-2-phenylindole (DAPI) (1:5000; Beyotime Biotechnology, Nantong, Jiangsu, China) for 5 min in the dark. Immunofluorescence images were obtained using a confocal fluorescence microscope (Leica, Solms, Germany).

### Evaluation of Neurological Function

The modified neurological severity score (mNSS) was used to evaluate the neurological status at 1, 3, 7, and 14 days after TBI (17). The mNSS is based on motor, sensory, balance, and reflex tests, with scores of 0 and 14 representing a healthy state and maximal deficit, respectively.

Motor coordination in mice was evaluated using the rotarod test (18). Briefly, the mice were trained over 3 days to remain for at least 5 min on a rotarod whose speed was gradually increased from 0 to 40 rotations/min; mice that were unable to meet this requirement were excluded from the experiment. The amount of time that a mouse was able to remain on the rod was evaluated at 1, 3, 7, and 14 days post-surgery.

Spatial learning and memory in mice were evaluated using the Morris water maze test as previously described (19, 20), with modifications. A circular tank filled with warm water that was rendered opaque by the addition of white lime was used. The pool was divided into four equal-sized quadrants each with a unique visual cue, and a 10-cm diameter platform was submerged 1 cm below the water surface in quadrant 1. On training days (14–18 days after surgery), the mice underwent four training trials per day in which the starting position was randomly switched between the four quadrants. A maximum time of 60 s was allowed for mice to locate the platform. If a mouse failed to do so within this time frame, it was manually guided to the platform and allowed to remain there for 15 s. On the test day (19 days after surgery), a probe trial was performed after removing the platform. The mice were then placed in the opposite quadrant and allowed to swim freely for 60 s. The latency to reach the former location of the platform and time spent in the target quadrant were recorded and calculated.

### Cell Culture

SH-SY5Y cells were obtained from the Stem Cell Bank, Chinese Academy of Sciences (Beijing, China) and cultured in Dulbecco's Modified Eagle's Medium (DMEM) (Gibco, Grand Island, NY, USA) containing 10% fetal bovine serum and 100 μg/ml penicillin/streptomycin at 37°C in a humidified incubator of 5% CO_2_ and 95% air.

### Cell Viability Assay

The viability of cells at different concentrations of serpina3k (50, 100, 150, 200, and 250 nM) was evaluated using the Cell Counting Kit (CCK)-8 assay (Dojindo, Kumamoto, Japan). SH-SY5Y cells were seeded in 96-well plates at a density of 1 × 10^4^/well and cultured overnight. After applying the different concentrations of serpina3k and culturing for 4 h, 10 μl of CCK-8 reagent was added to each well, followed by incubation for 1.5 h. The absorbance at 450 nm was measured with a spectrophotometer (Bio-Tek Instruments, Winooski, VT, USA).

### Cellular Model of Mechanical Injury

SH-SY5Y cells were subjected to SI to simulate TBI *in vitro*. The cells were seeded into BioFlex® Six-well Culture Plates (Flexcell International, Burlington, NC, USA) with collagen-coated silastic membranes at a density of 0.5 × 10^5^/cm^2^. Cells were subjected to biaxial SI with the Cell Injury Controller II instrument (Virginia Commonwealth University, Richmond, VA, USA), which released a 50-ms burst of nitrogen gas that caused a 7.5-mm downward deformation of the silastic membrane and adherent cells; this is analogous to the mechanical stress experienced by brain tissue during rotational acceleration and deceleration injury (3, 21).

### Experimental Cell Design

SH-SY5Y cells were randomly divided into control, SI+vehicle, and SI+A3K (50, 100, 150, 200, and 250 nM) groups and the optimal concentration of serpina3k was determined by evaluating cytotoxicity using the lactate dehydrogenase (LDH) release assay. Based on the results, we randomly divided the SH-SY5Y cells into three groups: control, SI+vehicle, and SI+A3K (optimal concentration). The vehicle or serpina3k solution was added immediately after SI, and the cells were continuously cultured until they were used for experiments that were repeated at least five times.

### LDH Release Assay

LDH release by SH-SY5Y cells was examined using a Cytotoxicity Detection Kit (Roche, Manheim, Germany) according to the manufacturer's instructions. Briefly, 100 μl of culture supernatant was collected from each group and transferred to a 96-well plate. After adding 100 μl of reaction solution to each well, the samples were incubated for 30 min at room temperature in the dark. The absorbance at 490 nm was then measured to determine the amount of released LDH.

### Detection of Apoptotic Cells

SH-SY5Y cell apoptosis was evaluated using flow cytometry measured by an Annexin V-FITC Apoptosis Detection Kit (Beyotime Biotechnology, Nantong, Jiangsu, China) according to the manufacturer's instructions. Briefly, the cells were resuspended in 195 μl of binding buffer containing 5 μl of Annexin V and 10 μl of propidium iodide (PI) and incubated for 15 min at room temperature in the dark. Apoptotic cells were analyzed using an Accuri C6 flow cytometer (BD Biosciences, San Jose, CA, USA). At the same time, the TUNEL assay was also adopted to detect apoptosis. In brief, after fixing with 4% paraformaldehyde and 0.3% TritonX-100, the cells were allowed to react with TUNEL buffer for 1 h at 37°C in the dark. Cell nuclei were stained with DAPI. The apoptotic cells were recorded using a confocal fluorescence microscope. The apoptosis rate (%) was calculated as the number of apoptotic cells/total number of cells in a field × 100%.

### Measurement of Intracellular Reactive Oxygen Species (ROS)

ROS in SH-SY5Y cells were detected using a fluorescent dichloro-dihydro-fluorescein diacetate (DCFH-DA) probe and DCFH-DA Assay Kit (Beyotime Biotechnology, Nantong, Jiangsu, China) according to the manufacturer's instructions. Briefly, 1 ml of 10 μM DCFH-DA solution was added to each well of a six-well culture plate, followed by incubation for 20 min at 37°C in the dark. After washing three times with DMEM, images were obtained using a fluorescence microscope. The mean fluorescence quantification was calculated as the total fluorescence in a field/all the cells in this field.

### Measurement of Mitochondrial Membrane Potential (ΔΨm)

The ΔΨm in SH-SY5Y cells was measured using a JC-1 Assay Kit (Beyotime Biotechnology, Nantong, Jiangsu, China) according to the manufacturer's instructions. Briefly, 1 ml of DMEM and 1 ml of JC-1 solution were added to a six-well culture plate and the cells were incubated for 20 min at 37°C in the dark. After washing twice with JC-1 buffer, JC-1 fluorescence was detected under the fluorescence microscope. When the ΔΨm is high, JC-1 forms an aggregate, which produces red fluorescence. However, when the ΔΨm is low, JC-1 exists as a monomer and generates green fluorescence. The decline of ΔΨm is a marker of apoptosis and the ratio of red fluorescence to green fluorescence, representing the change in ΔΨm, can be used as a detection index of cellular apoptosis.

### Western Blot Analysis of Cultured Cells

SH-SY5Y cells in different groups were collected and analyzed using western blot with primary antibodies against the following proteins: extracellular regulated protein kinases (ERK), phospho-ERK (p-ERK), P38, phospho-P38 (p-P38), B cell lymphoma (Bcl)-2, Bcl-2-associated X protein (Bax), caspase-3, and cleaved caspase-3 (all 1:1000; Cell Signaling Technology, Beverly, MA, USA); and β-tubulin, GAPDH, and β-actin (all 1:1000; Abcam, Cambridge, UK).

### Statistical Analysis

All data were shown as mean ± standard deviation (SD). Multiple-group comparisons were performed using one-way analysis of variance, and differences between two groups were evaluated using the unpaired Student's *t*-test. Statistical analyses were performed using SPSS v. 20.0 software (SPSS Inc., Chicago, IL, USA). *P* <0.05 and *P* <0.01 were considered to be statistically significant. Bar graphs were generated using GraphPad Prism v. 6.0 software (GraphPad, San Diego, CA, USA).

## Results

### Serpina3k Was Upregulated in the Peri-Contusion Area in Mice Following TBI

Changes in serpina3k protein levels between sham-operated tissue and peri-contusion areas at different time points post-TBI were evaluated using western blot. The fold change in serpina3k protein level relative to the sham group was 3.30 ± 0.45 at 6 h, 4.04 ± 0.20 at 12 h, 5.44 ± 0.52 at 24 h, 8.11 ± 0.25 at 3 days, and 4.02 ± 0.87 at 7 days post-TBI (*P* <0.01; Figures 1B,C).

### Serpina3k Inhibited the Loss and Apoptosis of Neurons in the Peri-Contusion Area Following TBI

**Figure 2:**
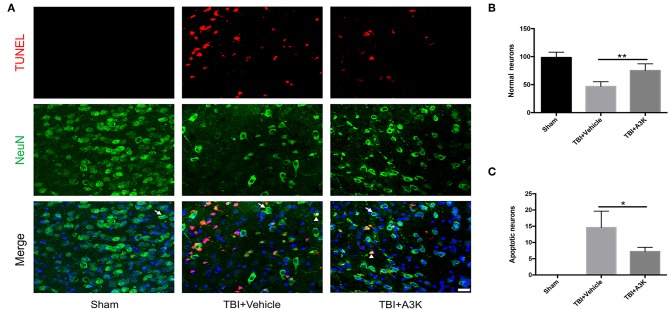
Serpina3k alleviated the loss and apoptosis of neurons at 7 days after TBI. **(A)** Representative images of TUNEL- and NeuN-positive cells in peri-contusion areas of brain tissue from mice in the sham, TBI+vehicle, and TBI+A3k groups. White arrows show normal neurons, white triangles demonstrate apoptotic neurons. Scale bar = 10 μm. Quantification of normal neurons **(B)** and apoptotic neurons **(C)**. Data represent the mean ± SD (*n* = 5 per group). *P <0.05, **P <0.01, TBI+A3K group vs. TBI+vehicle group.

After TBI, the large loss of normal neurons (white arrows) occurred and the number of apoptotic neurons (white triangles) increased in the peri-contusion area. However, serpina3k treatment reduced the loss of normal neurons (*P* <0.01; Figures 2A,B) and the number of apoptotic neurons (*P* <0.05; Figures 2A,C) relative to vehicle-treated mice at 7 days post-TBI.

### Serpina3k Reduced the Total and Each Neuron's Levels of Oxidative Stress in the Peri-Contusion Area After TBI

**Figure 3:**
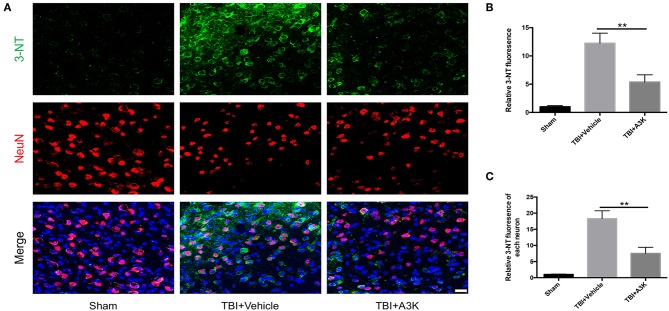
Serpina3k attenuated the total and individual neuron expression of 3-NT at 7 days after TBI. **(A)** Representative immunofluorescence images of 3-NT and NeuN in the peri-contusion areas of brain tissue from the sham, TBI+vehicle, and TBI+A3k groups. Scale bar = 10 μm. Quantified expression of total 3-NT **(B)** and each neuron's 3-NT **(C)**. Data represent the mean ± SD (*n* = 5 per group). **P <0.01, TBI+A3K group vs. TBI+vehicle group.

3-NT, an import index of oxidative stress and reactive oxygen species *in vivo* (22, 23), was used to assess the levels of oxidative stress at 7 days post-TBI. 3-NT was shown in green and NeuN, a neural marker, was shown in red. The total and each neuron's expression of 3-NT significantly increased following TBI-induced oxidative damage in the peri-contusion area. However, these increases were significantly ameliorated by treatment with serpina3k when administered 7 days after TBI (both *P* <0.01; Figure 3).

### Serpina3k Alleviated Neurological Deficits Caused by TBI

**Figure 4:**
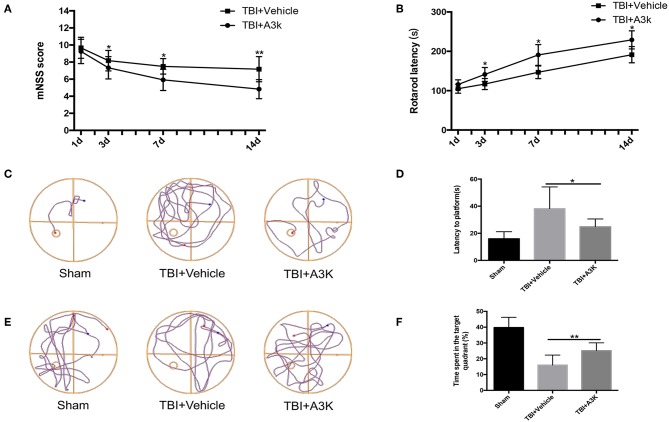
Serpina3k improved neurological function in mice following TBI. mNSS **(A)** and rotarod test results **(B)** at 1, 3, 7, and 14 days post-TBI in the TBI+vehicle and TBI+A3K groups. Representative traces **(C,E)**, and quantified time of first arrival at the platform **(D)** and percentage of time spent in the target (platform) quadrant **(F)** at 19 days after TBI in the sham, TBI+vehicle, and TBI+A3K groups. Data represent the mean ± SD (*n* = 12 per group). *P <0.05, **P <0.01, TBI+A3K group vs. TBI+vehicle group.

Mice in the TBI group showed neurological deficits. We evaluated the effect of serpina3k on functional recovery using the mNSS, rotarod test, and Morris water maze test. The mNSS was lower in the serpina3k treated mice compared with that in the vehicle-treated mice at 3, 7, and 14 days after TBI *(P* <0.05, <0.05, and <0.01, respectively; Figure 4A). In the rotarod test, the length of time that mice were able to remain on the rotarod was increased in the serpina3k treated compared with that of the vehicle-treated group at 3, 7, and 14 days after TBI (all *P* <0.05; Figure 4B). In the Morris water maze test, the results of latency to the platform and time spent in the target quadrant were improved in the serpina3k treated compared with that in the vehicle treated mice on the test day post-TBI (*P* <0.05 and <0.01, respectively; Figures 4C–F).

### Serpina3k Inhibited LDH Release and Apoptosis in SH-SY5Y Cells After SI

**Figure 5:**
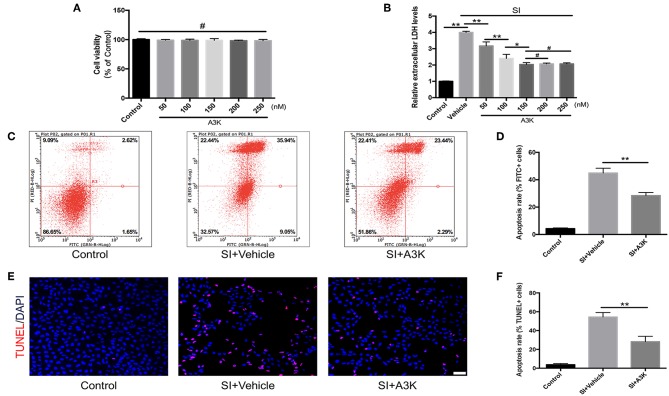
Serpina3k reduced damage to SH-SY5Y cells after SI. **(A)** Cell viability 4 h after treatment with different concentrations of serpina3k as determined using a CCK-8 assay. **(B)** Effect of different concentrations of serpina3k on the cytotoxicity of SH-SY5Y cells at 4 h after SI, as evaluated based on LDH release. Detection of apoptotic cells using Annexin V-FITC/PI double staining with flow cytometry analysis **(C)** and TUNEL staining **(E)**. Scale bar = 30 μm. Quantification of apoptosis rate (FITC+, **D**; TUNEL+, **F**) at 4 h after SI in the control, SI+vehicle, and SI+A3K groups. Data represent the mean ± SD (*n* = 5 per group). *P <0.05, **P <0.01; ^#^*P* > 0.05.

We investigated the mechanisms underlying the effects of serpina3k in SH-SY5Y cells that were subjected to SI to mimic contusive brain injury. We first evaluated the effect of exposure to different concentrations of serpina3k (50, 100, 150, 200, and 250 nM) for 4 h on cell viability with the CCK-8 assay and found no difference between groups (all *P* > 0.05; Figure 5A). In addition, LDH release—an apoptosis-associated index—was dramatically increased after SI compared with that in the control group (*P* <0.01) and was used to determine the optimal concentration of serpina3k at 4 h post-injury. With the increases in serpina3k concentration from vehicle to 150 nM, the release of LDH continuously reduced (*P* <0.01, <0.01, and <0.05, respectively), whereas no additional decreases were observed from 150 to 250 nM (both *P* > 0.05). We therefore used 150 nM serpina3k in subsequent experiments (Figure 5B). Annexin V-FITC/PI double-staining and flow cytometry were used to examine apoptotic cells in the control and SI groups. Annexin V-FITC+/PI- cells in the lower right quadrant showed early apoptotic cells and Annexin V-FITC+/PI+ cells in the upper right quadrant represented late apoptotic cells. Early and late apoptosis rates were obviously reduced following serpina3k compared with that following vehicle treatment at 4 h post-SI (28.41 ± 2.25% vs. 44.94 ± 3.47%) (*P* <0.01; Figures 5C,D). At the same time, consistent with the results of the Annexin V-FITC/PI double-staining, serpina3k treatment markedly reduced the percentage of apoptotic cells (TUNEL+) relative to the vehicle-treated group at 4 h after SI (28.23 ± 5.67% vs. 54.44 ± 4.71%) (*P* <0.01; Figures 5E,F).

### Serpina3k Suppressed Intracellular ROS Generation After SI

**Figure 6:**
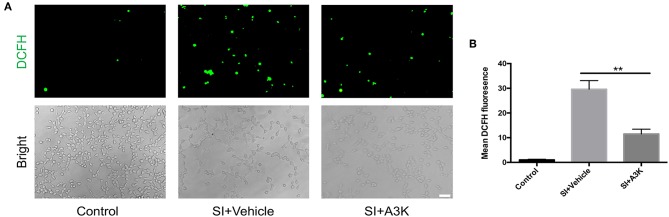
Serpina3k inhibited intracellular ROS generation in SH-SY5Y cells following SI. **(A)** Representative images of DCFH-DA fluorescence and bright field images of cells. Scale bar = 60 μm. **(B)** Quantification of mean fluorescence intensity at 2 h after SI in the control, SI+vehicle, and SI+A3K groups. Data represent the mean ± SD (*n* = 5 per group). **P <0.01, SI+A3K vs. SI+vehicle group.

After SI, ROS accumulation was increased in SH-SY5Y cells, as detected by DCFH-DA. However, mean ROS levels were decreased in serpina3k treated group relative to the vehicle-treated group at 2 h post-SI (*P* <0.01; Figures 6A,B).

### Serpina3k Alleviated the Changes in ΔΨm Caused by SI

**Figure 7:**
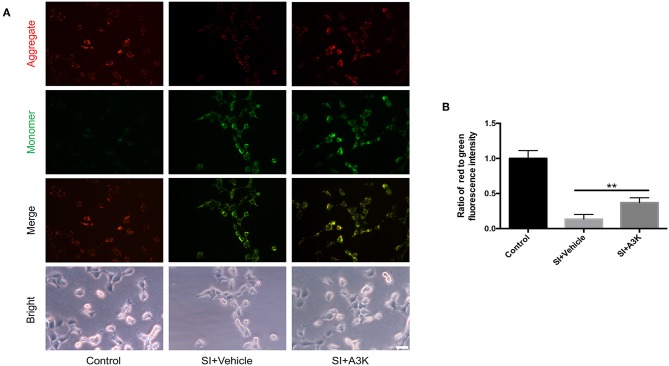
Serpina3k increased ΔΨm in SH-SY5Y cells after SI. **(A)** Representative images of JC-1 fluorescence reflecting ΔΨm and bright field images of cells. Scale bar = 40 μm. **(B)** Ratio of red to green fluorescence intensity at 2 h after SI in the control, SI+vehicle, and SI+A3K groups. Data represent the mean ± SD (*n* = 5 per group). **P <0.01, SI+A3K vs. SI+vehicle group.

The decline of ΔΨm in SH-SY5Y cells after SI was reflected by a change in JC-1 fluorescence from red to green. This effect was alleviated by treatment with serpina3k relative to vehicle treatment at 2 h post-SI (*P* <0.01; Figures 7A,B).

### Serpina3k Regulated Mitogen-Activated Protein Kinase (MAPK) and Mitochondrial Apoptosis Pathways After SI

**Figure 8:**
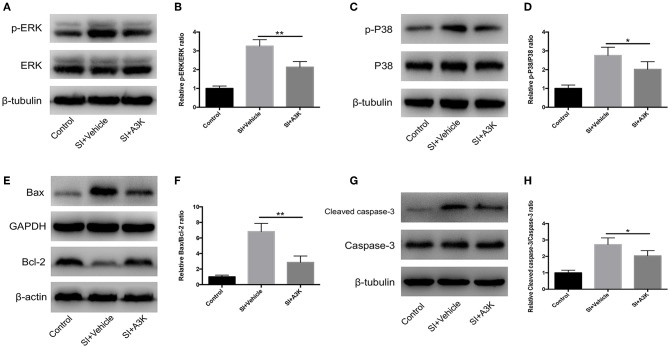
Serpina3k regulated MAPK and mitochondrial apoptosis pathways after experimental SI in SH-SY5Y cells. Representative western blots of p-ERK and ERK **(A)**, p-P38 and P38 **(C)**, Bax and Bcl-2 **(E)**, and cleaved caspase-3 and caspase-3 **(G)**. Quantified bar graphs of p-ERK/ERK **(B)**, p-P38/P38 **(D)**, Bax/Bcl-2 **(F)**, and cleaved caspase-3/caspase-3 **(H)** ratios at 2 h after SI in the control, SI+vehicle, and SI+A3K groups. β-tubulin, GAPDH, and β-actin were used as loading and internal controls. Data represent the mean ± SD (*n* = 5 per group). *P <0.05, **P <0.01, SI+A3K vs. SI+vehicle group.

We examined whether serpina3k targeted the MAPK and mitochondrial apoptosis pathways by analyzing the levels of associated proteins using western blot. We found that the p-ERK/ERK, p-P38/P38, Bax/Bcl-2, and cleaved caspase-3/caspase-3 ratios were increased at 2 h after SI, while these increases were attenuated by serpina3k treatment (*P* <0.01, <0.05, <0.01, and <0.05, respectively; Figures 8A–H).

## Discussion

TBI is associated with high rates of disability and mortality and is a major public health burden (24). Several TBI models have been established in mice in order to explore the underlying pathogenesis and evaluate potential therapies, including the fluid percussion injury (25), weight-drop injury (26), penetrating brain injury (27), and blast brain injury (28) models. In the present study, we used the CCI brain injury model owing to its ease of operation and high accuracy compared with that of other models. By manually controlling the impact parameters, we established a moderate TBI model as previously described (29).

Our results showed that endogenous levels of serpina3k protein were markedly elevated in the peri-contusion brain areas of mice after TBI. According to previous reports (13, 14, 30), serpina3k usually has anti-apoptotic functions in injury models. Administration of 0.5 mg/kg serpina3k twice daily to mice in the TBI group significantly reduced the loss and apoptosis of neurons in peri-contusion areas. After TBI, oxidative stress in areas of secondary damage is the major reason for apoptosis (31). In our study, we found that the expression of 3-NT in each neuron was obviously lower after serpina3k treatment than in vehicle-treated mice post-TBI, probably leading to the decrease in the number of apoptotic neurons. The observed improvements in mNSS and rotarod and Morris water maze test performance in mice with TBI treated with serpina3k provided further evidence that serpina3k mitigated neurological deficits, at least in part, by preventing the apoptosis of neurons.

To further determine the underlying mechanism of serpina3k effects following TBI, *in vitro* experiments were performed. In our study, the SI model, which causes similar damage to that of moderate TBI and has usually been used as an *in vitro* model of various neurological diseases, was applied to SH-SY5Y cells (32–34). Consistent with the results of our *in vivo* experiments, we found that the treatment of cells in the SI group with the optimal serpina3k concentration of 150 nM reduced early and late apoptosis, as detected using TUNEL staining and Annexin V-FITC/PI double-staining. The anti-apoptotic effect of serpina3k was further confirmed.

Apoptosis induced by TBI often starts with a decrease of ΔΨm and accumulation of ROS (21). A reduced normal mitochondrial population leads to abundant ROS, which further aggravates mitochondrial damage (35, 36). Excessive ROS in the cells can damage DNA, proteins, membrane lipids, and transcription factors, leading to the activation of several signaling pathways (15). According to previous studies (37, 38), the activation of the MAPK signal pathway is associated with the overproduction of ROS. Phosphorylation of ERK and P38 MAPKs induces apoptosis post-TBI (39). At the same time, endogenous mitochondrial apoptosis pathways are also activated after TBI (3). Bax and Bcl-2 are pro-and anti-apoptotic proteins that are present in the mitochondrial membrane; a high Bax/Bcl-2 ratio induces the release of cytochrome *c* from mitochondria into the cytoplasm, leading to caspase-3 cleavage and activation. The cleaved caspase-3 is the key player in the execution phase of apoptosis (15, 40). Our results indicated that serpina3k acted as an anti-oxidative agent and anti-apoptotic factor in SH-SY5Y cells following SI by suppressing intracellular ROS generation and inhibiting the decline of ΔΨm, and altering the p-ERK/ERK, p-P38/P38, Bax/Bcl-2 and cleaved caspase-3/caspase-3 ratios.

Our study has some limitations. First, the distribution of exogenous recombinant serpina3k in various cell types of the brain tissue could not be detected. A different tag needs to be added to the recombinant protein for future experiments. Second, although SH-SY5Y cells exhibit some features of neurons (21), primary cultured neurons might be more appropriate for investigating endogenous mechanisms after injury. Finally, the heparin-binding and active sites of serpina3k, which play a role in different signaling pathways, have been shown to be responsible for its dual activities in apoptosis (9); this should be examined in greater detail in order to clarify the mechanism of action of serpina3k.

## Conclusion

In conclusion, our results show that serpina3k enhances the recovery of neurological function following TBI by acting as an anti-oxidative agent to decrease the number of apoptotic neurons in mice. Subsequently, it was also shown that serpina3k could protect the SH-SY5Y cells by attenuating intracellular ROS generation, changes in ΔΨm, and regulation of MAPK and mitochondrial apoptosis pathways after SI. Thus, serpina3k treatment may be a promising therapeutic strategy for improving outcomes following TBI.

## Data Availability Statement

All datasets generated for this study are included in the article/supplementary material.

## Ethics Statement

The animal study was reviewed and approved by Institutional Animal Care and Use Committee of Shanghai Jiao Tong University.

## Author Contributions

HT, SC, JD, and GY designed the study. HC, FY, WW, and YJ performed the experiments. DY and YF collected and analyzed the data. YJ and DY drafted and edited the manuscript. All authors read and approved the final manuscript.

### Conflict of Interest

The authors declare that the research was conducted in the absence of any commercial or financial relationships that could be construed as a potential conflict of interest.
